# Standing with our medical colleagues

**DOI:** 10.1192/bjb.2020.67

**Published:** 2020-08

**Authors:** Sarah Castle

**Affiliations:** ST6 Doctor in Child and Adolescent Psychiatry, Tavistock and Portman NHS Foundation Trust, UK; email: sarah.castle@yahoo.co.uk

Like many of us I am increasingly concerned about the possible effects of coronavirus on myself, my family and our community and patients. With family in Italy, I am mindful of the need there for other medical professionals to be brought in to work alongside their acute medical colleagues. This has included those more distant from acute medical work now, such as pathologists and even in some cases psychiatrists.

Little has been spoken about our role in regards to COVID-19 should we see similar scenes here in the UK and what our possible involvement might be. It would be easy to adopt a ‘protectionist’ attitude and leave the messy stuff to ‘real doctors’.

As a psychiatrist, I am a doctor of the mind and body. Of course, I would never advocate working beyond our professional competencies; however, I feel we have a strong duty to stand beside our medical colleagues be it pushing trolleys, taking blood or sweeping floors if the need arises. We spend so much time and energy trying to reduce the splitting that occurs between ‘medicine’ and ‘psychiatry’; surely we cannot hide behind this very split now when our colleagues and indeed our communities may need us if an outbreak worsens to the degree that many fear. Surely then we must stand together as one profession.

